# The Promise and the Challenge of Technology-Facilitated Methods for Assessing Behavioral and Cognitive Markers of Risk for Suicide among U.S. Army National Guard Personnel

**DOI:** 10.3390/ijerph14040361

**Published:** 2017-03-31

**Authors:** Brian R. W. Baucom, Panayiotis Georgiou, Craig J. Bryan, Eric L. Garland, Feea Leifker, Alexis May, Alexander Wong, Shrikanth S. Narayanan

**Affiliations:** 1 Department of Psychology, University of Utah, Salt Lake City, UT 84108, USA; craig.bryan@psych.utah.edu (C.J.B.); feea.leifker@psych.utah.edu (F.L.); alexis.may@psych.utah.edu (A.M.); alexander.wong@psych.utah.edu (A.W.); 2 Department of Electrical Engineering, University of Southern California, Los Angeles, CA 90089, USA; georgiou@sipi.usc.edu (P.G.); shri@ee.usc.edu (S.S.N.); 3 National Center for Veterans Studies, University of Utah, Salt Lake City, UT 84108, USA; 4 Department of Social Work, University of Utah, Salt Lake City, UT 84108, USA; eric.garland@socwk.utah.edu

**Keywords:** suicide risk, military, Behavioral Signal Processing, cognitive assessment

## Abstract

Suicide was the 10th leading cause of death for Americans in 2015 and rates have been steadily climbing over the last 25 years. Rates are particularly high amongst U.S. military personnel. Suicide prevention efforts in the military are significantly hampered by the lack of: (1) assessment tools for measuring baseline risk and (2) methods to detect periods of particularly heightened risk. Two specific barriers to assessing suicide risk in military personnel that call for innovation are: (1) the geographic dispersion of military personnel from healthcare settings, particularly amongst components like the Reserves; and (2) professional and social disincentives to acknowledging psychological distress. The primary aim of this paper is to describe recent technological developments that could contribute to risk assessment tools that are not subject to the limitations mentioned above. More specifically, Behavioral Signal Processing can be used to assess behaviors during interaction and conversation that likely indicate increased risk for suicide, and computer-administered, cognitive performance tasks can be used to assess activation of the suicidal mode. These novel methods can be used remotely and do not require direct disclosure or endorsement of psychological distress, solving two challenges to suicide risk assessment in military and other sensitive settings. We present an introduction to these technologies, describe how they can specifically be applied to assessing behavioral and cognitive risk for suicide, and close with recommendations for future research.

## 1. Introduction

Suicide was the 10th leading cause of death for Americans in 2015 [1], killing over 44,000 individuals. Despite increases in prevention and research funding, suicide rates have been steadily climbing over the last 25 years [2]. This trend has been mirrored and amplified among U.S. service members, a group that has historically carried a lower risk for suicide [3]. Suicides among U.S. service members have more than doubled since 2002. In 2008 the historical trend for a lower suicide rate among military personnel ended when the active duty military suicide rate surpassed that of the demographically matched U.S. general population (20.2 per 100,000 vs. 19.2 per 100,000) for the first time in known history [4]. Suicides have increased across all branches of the U.S. military, but have increased most dramatically in the Army. In response to steadily rising suicide rates among U.S. military personnel and veterans, researchers, clinicians, policy-makers, and military leaders have responded with an overwhelming and concerted effort to reverse these trends [5]. Suicides have been especially high in the Army National Guard and Reserve components, suggesting this group is an especially high-risk subgroup of the military [6]. Although Soldiers in the National Guard are almost twice as a likely to die by suicide as their active duty peers, however, very few studies have been conducted in this military component.

Suicide prevention efforts in the National Guard are hindered by several important factors. First, because National Guard personnel tend to be more geographically dispersed than their active duty counterparts and are often located far from military medical facilities, access to professional services is limited. Spouses and partners therefore play a central role in the care and support of National Guard Soldiers. Although marriage is a well-established protective factor for suicide [7], relationship problems can serve as a risk factor for suicide across populations [8,9,10]. Unfortunately, little is known about the specific relationship processes and dynamics that can make the relationship protective in some couples and a risk factor in others. Therefore, suicide prevention efforts that focus on relationship functioning may not be targeting the aspects of the relationship dyad that are most directly related to suicide risk. Second, suicide prevention screening primarily occurs within medical and military settings using self-report methods. As such, it occurs relatively infrequently and is highly vulnerable to reporting bias. That is, National Guard service members at risk of suicide may be less likely to disclose their suicidal thoughts due to fears about job security, security clearance, or general stigma [11]. Finally, suicide prevention efforts have been hampered by a general dearth of information regarding observable and reliable indicators of imminent risk for suicide, or suicide warning signs. As recent meta-analyses have shown, most of the risk factors commonly assessed (e.g., depression, substance use, or hopelessness) do little to identify individuals at risk of suicide attempts or deaths [12,13]. For example, while those experiencing symptoms of depression are at an increased risk of suicide, the vast majority of those with depression will not attempt or die by suicide. Thus, considerable confusion exists regarding when and under what circumstances certain behaviors signal the emergence of acute risk for suicide and when those same behaviors do not. 

## 2. Conceptualizing the Association of Marriage, Relationship Problems, and Suicide

Spouses of soldiers occupy a unique position in the constellation of factors associated with suicide, as empirical evidence suggests that marriage can serve as both a protective factor and a risk factor. On the one hand, spouses can be a strong source of support and assistance during times of stress; on the other hand, relationships and marriages are often sources of stress that precipitate crises. Research has generally found that married individuals have significantly lower suicide rates than individuals who have never married, individuals who are divorced, and individuals who are widowed [7]. Findings regarding the association of marital status to suicide risk among soldiers are less straightforward, however. Among first-term active duty soldiers, those who are married are less likely to have a history of suicide ideation and suicide attempt [14]; but, when considering the Army as a whole, active duty married soldiers are more likely to make a suicide attempt [15]. Within the subgroup of active duty soldiers who are married, those who have never deployed are more likely to die by suicide [16], which counters the common assumption that suicide in the military is often the consequence of relationship dissolution in the aftermath of repeated deployments. In contrast to active duty soldiers, National Guard soldiers who are married are less likely than unmarried soldiers to die by suicide [17], although the ending of a significant relationship is associated with markedly increased risk for suicide ideation and attempts [18]. Similar findings have been reported in nonmilitary samples. Boardmann and colleagues [19] reported that the protective effect of marriage is largely confined to married couples who are not separated from their spouses; those who are married but separated from their spouses have a similar suicide rate to unmarried individuals. Similarly, Barstad [20] found that separation from one’s spouse is a better indicator of suicide risk than divorce, a finding that aligns with research supporting relationship discord as a risk factor for suicide. 

Epidemiological data indicate that up to 80% of suicide deaths among active duty and National Guard soldiers are associated with at least one identifiable life stressor, of which the most frequently-occurring are relationship problems [21,22]. Recent findings further indicate that relationship problems are the most frequently-endorsed stressors occurring within the 24 h immediately preceding active duty soldiers’ suicide attempts [8]. Among active duty soldiers who attempted suicide, the most common relationship problems included arguments and conflicts (58%), feeling criticized or put down by another person (49%), and feeling rejected (32%; [8]). Research has subsequently found that suicidal active duty soldiers who experience persistent relationship problems are more likely to have made multiple suicide attempts and to experience extended suicidal episodes that last for up to six months without resolution [23]. According to the fluid vulnerability theory of suicide (FVT; [24]), these findings may be explained by differentiating between chronic (i.e., persistent) and acute (i.e., dynamic) variables. Specifically, chronic relationship problems marked by persistent criticism and perceived rejection can have a more pronounced deleterious effect on an individual partner’s sense of identity (e.g., “I’m a failure”, “I’m unworthy of love”) as compared to shorter-duration and relatively circumscribed relationship conflicts. Chronic relationship problems would, therefore, contribute to stable risk factors that persist for comparatively long periods of time. Among couples with elevated levels of stable risk factors, acute flare ups related to relationship issues such as fights or arguments are compounded and even magnified, such that acute conflicts (and their attendant emotional distress and dysphoria) last longer and are harder to resolve for the couple. Couples characterized by chronic relationship problems are therefore more likely to experience more severe and long-lasting episodes of suicide risk within the context of relationship strain. 

In contrast, suicide risk may be lower in couples who engage in supportive and collaborative behaviors while discussing relationship problems. The ability to use such behaviors during relationship conflict is likely indicative of a general ability to work together as a unit to solve problems, to down-regulate stress, and to maintain an emotional connection even when upset about problems. Such behavioral processes are reflected in both spouses’ behaviors as well as in the way that they respond to one another’s behaviors. A strong relationship may therefore decrease chronic risk for suicide, as well as buffering some of the possible effects of acute relationship conflicts. Improved understanding of these factors could lead to the refinement of programs and treatments designed to prevent suicide through enhanced relationship quality.

## 3. Limitations to Existing Suicide Risk Detection Methods

Arguably the most pressing problem of suicide prevention is accurate and reliable identification of at-risk individuals. Within the Army National Guard, suicide risk screening typically occurs on a recurring but relatively infrequent schedule using highly face-valid items that directly ask about suicidal thoughts and behaviors. Further, although suicide prevention screening has been implemented widely within the Army National Guard, existing screening methods do not always positively identify high-risk soldiers who will make a suicide attempt because: (a) the timing of the screening does not necessarily coincide with the emergence and course of an active suicidal episode and/or (b) soldiers may be motivated to conceal or minimize their risk. Research indicates that the majority of individuals who make a suicide attempt or die by suicide are “missed” by current screening methods or because they do not disclose their suicidal intent to others [25,26]. Despite widespread implementation of universal depression and suicide prevention screening across military medical clinics and routine health assessment, U.S. military data mirror this trend. Only two-thirds of active duty soldiers who die by suicide access some form of medical, mental health, or other support service in the 90 days preceding their deaths, and only 25% who die by suicide disclose suicidal thoughts or intent prior to their deaths [22]. Among National Guard soldiers, approximately one-third with current suicide ideation deny these thoughts on screening measures that are not anonymous [9]. This is a critical issue for the National Guard because soldiers who are missed by screening tools are unlikely to receive potentially life-saving interventions (e.g., brief cognitive behavioral therapy [27]).

**Figure 1:**
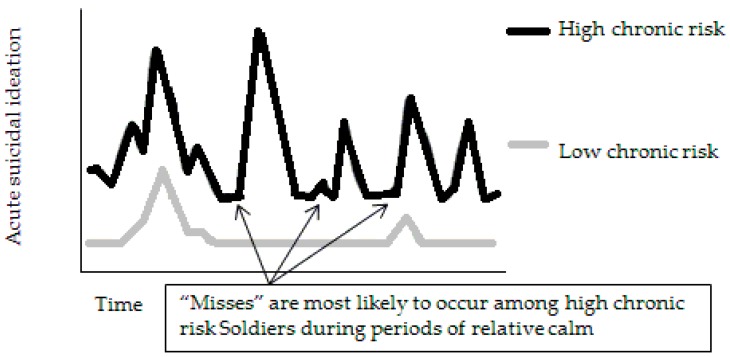
Variability in risk for suicide over time in high and low chronic risk individuals.

According to the FVT, the accuracy of detecting at-risk individuals is improved by considering both chronic and acute risk dimensions simultaneously. Because existing screening methods within the National Guard primarily focus on the acute dimension of risk (e.g., current depression, suicide ideation during the past week), the highest risk soldiers may paradoxically be the soldiers most likely to be missed by existing screening methods. This problem is graphically displayed in Figure 1, which plots fluctuations in suicidal risk over time for two different soldiers: a high chronic risk soldier (the black line) and a low chronic risk soldier (the grey line). The high chronic risk soldier is at greater risk for suicide over time relative to the low chronic risk soldier. However, if the high-risk soldier is screened during a period of relative calm (i.e., while in a “valley” between “peaks” of distress), he or she would deny current suicidal ideation and consequently look no different from the low risk soldier. The high-risk soldier would therefore be “missed.”

In contrast to medical professionals and military leaders and peers, all of whom have only periodic contact with the at-risk soldier (e.g., at monthly drill), spouses may be much more likely to notice indicators of elevated acute risk that might otherwise remain undetected by the military system. Furthermore, partners often serve as the primary source of social support for at-risk military veterans [28], but to date little is known about why some partners are more effective at providing support than others. Improved understanding of relationship dynamics that contribute to better and worse outcomes for at-risk soldiers could provide a valuable new avenue for differentiating high and low chronic risk individuals as well for recognizing periods of increased acute risk.

## 4. Relationship Conflict Behavior, Behavioral Signal Processing, and Warning Signs for Suicide

Another significant barrier to effective suicide risk detection prediction is figuring out when risk factors for suicide indicate emerging risk and when they do not. For example, although relationship problems are a well-established risk factor for suicide, most soldiers who experience relationship conflict do not make a suicide attempt. However, it is likely that periods of particularly intense relationship distress in chronically distressed couples coincide with periods of increased acute risk for suicide. Based on observational research, researchers have identified consistent behavioral markers of relationship distress. Distressed couples are often more negative [29,30], critical [29,31], aversive [32], and hostile and aggressive [29,32,33] compared to nondistressed couples. Researchers also consistently tend to find that distressed couples engage in specific patterns of behavior such as demand/withdraw and negative reciprocity more frequently than non-distressed couples [34,35,36].

In addition to identifying behavioral patterns indicative of relationship distress, researchers have identified unique behavioral characteristics of couple interactions in which one or both partners are depressed, suggesting it may be possible to identify behavioral markers unique to psychpathology and/or risk for suicide. Similar to interactions between couples with low marital satisfaction, interactions between couples with a depressed partner tend to be less positive and more negative, including increased hostility, negative projections about the future, withdrawal, reassurance seeking, and self-denigration compared to interactions among non-depressed couples [37,38,39,40,41]. This behavior may also be evident in the linguistic patterns of depressed individuals; during conversations with opposite sex strangers, depressed individuals show greater frequency of prolonged hesitations and silence, tend to be quieter, and are more monotone than non-depressed individuals [39,42]. Furthermore, the unique contributions of depression on conflict behaviors can be identified even after taking into account the level of relationship satisfaction [43], suggesting predictors unique to dysphoria or other acute signs of suicidality may be identified.

While no research to date has examined behavioral predictors of suicidal thoughts or behaviors from communication behaviors during couple interactions, less self-disclosure has been found to be characteristic of interactions between couples with a depressed partner [37]. Self-reported problems with self-disclosure are, in turn, associated with increased suicidal thoughts and behaviors [44]. Additionally, physical and psychological intimate partner violence victimization has been linked with suicidal thoughts and behaviors [9,45], suggesting that particularly hostile or aggressive conflicts may have the potential to be an acute predictor of suicide.

This gap in assessment tools can be filled by new technologies that can support the measurement, analysis, and modeling of behavior with unprecedented efficiency, scalability, reliability, and flexibility. One such technology is Behavioral Signal Processing (BSP [46]). BSP refers to a suite of computational technologies and tools that enable automated measurement of people’s behaviors during recorded conversations. The primary goal of BSP is to take a continuous audio recording of a conversation, extract mathematical quantities (called features) from that recording, and use the extracted features to measure and model behavior. In this regard BSP is similar to other signal processing methods commonly used in the behavioral sciences. For example, psychophysiological research uses signal processing to derive summary indices of physiological activity from continuous recordings of electrical activity (e.g., calculating heart rate from an electrocardiogram). Similarly, BSP uses an audio recording of a conversation to model how much blame, negative affect, acceptance, etc., was expressed during a conversation [47]. BSP achieves this aim through a series of steps that include automatically determining when someone is talking (i.e., voice activity detection), determining who is talking (i.e., diarization), creating an automated transcript (i.e., automatic speech recognition), extracting acoustic and linguistic features, and using those features to model target behavioral constructs.

Recent research has focused on the creation and development of BSP methods for dyadic interaction in general, with a specific emphasis on applying them to couples’ discussions of relationship problems as well as to conversations between suicidal soldiers and their therapists. This work has resulted in numerous algorithms for automatically measuring well-established behavioral markers of relationship distress during couple conversations about relationship problems [47] as well as discovery of behavioral and emotional processes associated with acute emotional distress during couple conversations and during soldier-therapist interactions [48,49]. For example, one particularly important indicator of acute distress is the degree to which one person is emotionally responsive to the other. This process is referred to as “entrainment” and higher levels of vocal entrainment (i.e., being more vocally “matched”) have consistently been found to be associated with lower levels of acute distress during both couple and soldier-therapist interactions [48,50,51].

These findings illustrate why BSP is an ideal approach for creating behavioral markers of “suicide warning signs” during couple interactions. First, BSP generates behavioral markers that are psychologically meaningful and interpretable. Second, there are proven BSP algorithms for measuring behaviors that are likely related to acute suicide risk, namely those associated with relationship distress and those associated with acute emotional distress in conversations involving a suicidal soldier. Third, BSP algorithms developed in one kind of interaction can be adapted for use in other kinds of interactions. For example, the method for measuring entrainment was developed using relationship problem conversations and was readily extended to soldier-therapist interactions. Finally, once BSP algorithms are developed, they can be repeatedly used to assess future couple interactions, thus tracking fluctuations risk levels over time. This set of features and benefits demonstrates that (a) BSP is a proven technology for measuring and modeling behavioral markers of relationship and emotional distress, (b) the existing foundational technologies can be further enhanced and refined to increase our ability to accurately and efficiently recognize changes in acute risk for suicide in a timely manner, and (c) because these technologies are computer-driven, they could potentially be accessed outside of healthcare settings (e.g., at home) via the internet.

## 5. Mechanics of Measuring Behavior with BSP

Understanding the procedural details of measuring behavior with BSP is important for clarifying their potential for aiding in the detection of risk for suicide outside of healthcare settings. While spouses and romantic partners are uniquely positioned to notice acute warning signs of risk for suicide given their daily or frequent interactions with their partner, they are unlikely to have any training in assessing risk for suicide. Furthermore, depressed and suicidal individuals are less likely to directly disclose their state of mind [37,44]. Thus tools that are simple to use, that do not ask partners or spouses to make decisions or judgments that require training, and that are not dependent on direct self-disclosure are likely to be particularly useful. Below we describe the mechanics involved in creating behavioral markers using BSP. BSP also involves additional data processing steps prior to generating behavioral markers; an overview of these steps is presented in Appendix A.

BSP can be used to measure two broad classes of behavior. The first class, automated observational coding markers, replicates behavioral judgments made by highly trained behavioral coders (i.e., negative emotions identified by trained people). Automated observational coding markers include well-established conflictual and supportive communication behaviors that are widely used in relationship science to measure behavior during couple interactions (see [36] for a review). Example conflictual behaviors that can be identified via automated observational coding markers include anger, blame, and contempt, and example supportive behaviors include acceptance, affection, soliciting the spouse’s perspective, and offering emotional support. These communication behaviors are typically measured by having trained research assistants watch a recording of a conversation and scoring the frequency, intensity, or severity of different behaviors based on rules defined in a coding manual. Examples of such coding systems include the Couples Interaction Rating System [52] and the Social Support Interaction Rating System [53]. BSP uses acoustic (i.e., qualities of sound) and lexical (i.e., qualities of the words that are spoken) features to generate scores that match those that would be generated by a trained human rater. In other words, BSP is able to determine what a human rater would say from the combination of features that describe what words were said and how those words were said. For example, Black and colleagues [47] used BSP methods to generate observational coding values that correlate 0.60–0.80 with human ratings of the conversations, depending on the behavior being coded. Recent developments have extended these efforts by incorporating artificial intelligence techniques resulting in increased agreement with human ratings (e.g., [54,55]).

The second class of behavioral measures generated by BSP, feature-derived behavioral markers, refers to measures of communication and affective behavior that are mathematical summaries of different aspects of vocal behavior, which are based directly on signal features themselves rather than on behaviors defined by a coding manual (i.e., computer-driven). This kind of behavioral measurement is conceptually similar to signal processing derived measures used in psychophysiological research. For example, heart rate is the number of R waves that occur during 60 s, where R waves are defined by the shape and timing of the electrocardiogram (ECG) signal waveform. In much the same way, the fundamental frequency of a speech waveform is a measure of the lowest frequency harmonic in an individual’s voice and is defined by the shape and timing of the speech signal waveform. Both measures index some aspect of the frequency content of a complex waveform. Fundamental frequency is just one example of numerous feature-derived markers that can be generated using BSP. Feature-derived markers are particularly valuable when combined with automated observation coders markers because feature-derived markers measure aspects of behavior that are too complex to measure with observational coding (e.g., those that occur over multiple timescales and/or that involve a large number of behavioral cues).

Of the large number of candidate feature-derived markers, there are three specific categories of markers that are particularly likely to be useful for detecting short-term fluctuations in suicide risk: entrainment, affective markers, and saliency.

Spouses are well-attuned to each other’s behaviors. Fluctuations in suicide risk are, therefore, likely to be reflected in the inter-relatedness of a soldier’s behavior and his/her spouse’s behavior. This “matching” or attunement between the soldier and spouse is referred to as entrainment. Vocal entrainment during a couple’s conversation is related to a range of behaviors and emotions, such as demand/withdraw behavior and positive and negative affect [56]. Building on these findings, recent work has focused on devising direct similarity measures between the vocal feature spaces of romantic partners using principal component analyses and has demonstrated further robust results in characterizing the latent vocal behavior similarity as well as its utility for predicting couple behavior [57,58].

The rich emotional information contained in speech and spoken language can be computationally modeled using both verbal and non-verbal information. The end product of this modeling is referred to as affective markers. Vocal cues, including speech, spoken language, and nonverbal vocalizations (e.g., scoffing, laughter, etc.) and disfluency patterns, carry rich information about emotions. Deciphering and interpreting these rich cues requires computational modeling at multiple contextual scales and levels, from varying levels of linguistic abstraction (e.g., voice quality, phonemic, lexical, prosodic, syntactic, discourse) to the level of the larger social-emotional and environmental context. Relevant research includes early work on detecting affect in spoken dialogs (e.g., [59]) and gestures and body posture (e.g., [60]), using primitives of affect (e.g., [61]), and developing real-time systems (e.g., [62]).

The third and final feature-derived marker that is likely to be useful in detecting risk for suicide is saliency. Saliency refers to how prominent or noticeable a behavioral marker is relative to other behavioral markers. Saliency indices can be estimated using both bottom-up and top-down approaches. Bottom-up saliency refers to low level signal features (e.g., changes of sound energy) while top-down saliency refers to changes in high-level behaviors (e.g., sudden change from positive to negative attitude of the speaker). Bottom-up saliency detection is typically performed via fusion of low-level spectro-temporal speech signal features including energy change, periodicity/harmonicity, spectral component variation, and spectral fluctuation [63]. In contrast, top-down saliency detection can be estimated using multiple instance learning [64]. Multiple instance learning can be regarded as a generalized supervised learning paradigm, in which only sets of examples, and not single examples themselves, are associated with observational codes. The examples are referred to as “instances”, while the labeled datasets are called “bags”. For example, a couple’s conversation could be viewed as a bag, and utterances within the conversation as instances. Conventionally, a negatively labeled bag is assumed to contain only negative instances, while a positive bag should contain at least one positive instance. Diverse Density Support Vector Machines can be used to robustly predict high-level behavioral codes from low-level lexical and intonation features [65].

In sum, the combination of automated observational coding markers and feature-derived behavioral markers generates a very thorough and detailed quantitative description of behavior during human interactions. These two types of behavioral markers allow for measuring behaviors that are likely to be associated with both chronic and acute dimensions of risk for suicide by virtue of their association with variables that are known to be associated with increased risk for suicide (e.g., relationship distress, psychological disorders). Feature-derived behavioral markers also allow for exploration of a large number of additional markers that could provide valuable information about short-term fluctuations in risk for suicide. The value of feature-derived behavioral markers is not only the number of potential markers but also their ability to capture important aspects of interaction that are too complex for humans to reliably code (e.g., the most salient periods of a 10-minute conversation).

## 6. Cognitive Bias and Suicide

Similar to BSP generated behavioral markers, performance-based behavioral measures of implicit cognitive biases and relationship functioning are well suited for development into a tool that could be used by spouses to detect short-term increased risk outside of healthcare settings. These measures are computer-administered and can be completed over the internet (e.g., [66]). 

A growing body of research suggests that cognitive processes such as these may be best assessed through a combination of implicit and explicit measures. For example, recent empirical evidence suggests that measures of implicit relationship satisfaction explain a large amount of unique variance in relationship health and stability above and beyond that which is explained by self-report measures of explicit relationships satisfaction [67]. Likewise, research suggests that implicit (or non-conscious) measures of intrapersonal cognitive processes provide significantly improved predictive validity of suicide risk above and beyond that which is attainable using explicit measures of other clinical risk factors [68,69].

Conceptually, implicit (or non-conscious) automatic processing of suicide-related cognitions occur during acute suicidal episodes. These processes become manifest in a fixation of attention on suicide-related stimuli (i.e., suicide attentional bias) and an association of one’s sense of self with the idea of death (i.e., suicidal implicit association). Cognitive biases are associated with preoccupation with negative emotional content and an obsessive focus that is associated with amplified and prolonged dysphoric moods and self-destructive behaviors [70]. Thus, a soldier at risk for suicidal behavior may become preoccupied with thoughts of his or her own death triggered by self-referential thoughts or attentional hypervigilance for external cues associated with death. Because these thoughts can occur with little to no conscious awareness, soldiers with increased risk for suicide may be unable to accurately report their risk to others. This decreased ability arises from the unconscious nature of many motivations and behavioral impulses [71,72] and the deleterious effect of psychological distress on executive functions [73]. These two factors can impair at-risk individuals’ insights into their thoughts and feelings about themselves and their relationships. The use of both explicit and implicit measures of intrapersonal cognitive processes to assess suicide risk provides incremental benefit as compared to the use of explicit measures only. 

Implicit suicidal beliefs can be measured using the Suicide Implicit Association Test (S-IAT; [69]). The S-IAT is an approximately 10-min computer-based reaction time test that asks individuals to classify words that appear in the middle of the computer screen into concept and attribute categories by pressing either a left or right key on a computer keyboard. In the S-IAT, participants classify words as quickly as into the construct of “death” and “life” with the attributes of “me” and “not me”. In the second section of the test, participants are asked to classify words in response to both “death” and “me” or “life” and “not me.” Reaction time is measured and recorded in milliseconds, and then analyzed using a standard IAT scoring algorithm. Suicide attentional bias can also be measured with a computer-administered, performance-based task known as a dot probe task [74]. During dot probe tasks, participants are briefly presented with pairs of stimuli, one emotional and one neutral, which then disappear. One of the stimuli is subsequently replaced by a target probe (a dot) whose location is identified by a button press. The reaction time to identify the location of the dot is then recorded. Because attention is biased by emotional information, reaction times tend to be faster for stimuli that match an individual’s current emotional concerns and motivational drives than for neutral stimuli, and thus an index of attentional bias can be computed from the dot probe by subtracting the reaction time to probes replacing emotional stimuli from the reaction time to probes replacing neutral stimuli. Suicide attentional bias could be measured by adapting existing dot probe task procedures to compare reaction times to target probes replacing neutral words (table, brown) with reaction times to probes replacing suicide-related words (death, kills) and negative words (angry, frown). Equal proportions of neutral/suicide-related and neutral/negative would be presented in two separate blocks where each pair of stimuli are presented for either 200 ms or 2000 ms, with one word positioned above and the other below a fixation cross. Faster reaction times to probes replacing suicide-related words compared to probes replacing neutral words would be indicative of a suicide attentional bias. Suicide-specific attentional bias effects above and beyond those of general attentional biases towards negative emotional stimuli could be determined by computing attentional bias scores for negative word stimuli relative to neutral word stimuli and comparing this bias score to the one for suicide-related words. Though this method of assessing suicide-specific attentional bias draws on widely used dot probe methods for assessing attentional bias in a wide range of psychological disorders, the psychometric properties of this suicide-specific adaptation of the dot probe task would need to be rigorously tested prior to their implementation for detecting suicide risk in National Guard personnel. Such tests would ideally be conducted using a National Guard sample in order to establish the task’s validity in the primary population of interest. 

Methods for measuring implicit beliefs regarding relationship satisfaction have also been developed. Specifically, the partner-focused go/no-go association task (Partner GNAT; [67]) asks participants to sort words into either good, bad, or spouse-related categories. For each four block of trials, participants are given a target category and asked to press a button if a word belongs to the target category and to not press a button if the word does not belong to the target category. Target categories include good, bad, good or spouse-related (i.e., spouse-good), and bad or spouse-related (i.e., spouse-bad). Words are presented one at a time for 600 ms followed by a 400 ms interstimulus interval before presentation of the next word. Words for the spouse-related category are provided by each participant before beginning the task. A final D score is calculated separately for the spouse-good and the spouse-bad trials as the difference between the standardized hit rate and the standardized false alarm rate for each type of trial. Higher spouse-good scores indicate greater implicit relationship satisfaction whereas higher spouse-bad scores indicate lesser implicit relationship satisfaction. Similar to the suicide-specific dot probe task, the psychometric properties of the Partner GNAT would need to be tested in samples of suicidal individuals and National Guard personnel prior to their implementation for detecting suicide risk in National Guard personnel.

In summary, implicit measures of suicide risk and relationship functioning do not require the direct endorsement or acknowledgement of psychological distress or suicidal ideation, intent, or planning. They also do not require face-to-face interaction with another individual. These qualities make implicit measures well-suited to soldiers who may be reluctant to talk with another person about their thoughts or feelings, or who may be reluctant to take a highly face-valid test that is obviously measuring some aspect of risk for suicide. 

## 7. Empirical Evaluation of BSP-Based Measures of Behavior and Computer-Based Measures of Implicit Cognitive Bias to Detect Risk for Suicide

BSP and computer-administered tests of cognitive bias have great potential to detect behavioral and cognitive markers of chronic and acute risk for suicide. However, there are several empirical tests that these methods must pass in order to demonstrate their true value in assessing risk for suicidal behavior: (1) the ability to discriminate amongst chronic risk groups; (2) the ability to detect time-varying risk within different chronic risk groups; and (3) the ability to pass tests 1 and 2 with behavioral and cognitive data collected under differing circumstances and of variable quality.

A first empirical test that these methods must pass is the ability to accurately differentiate couples based on preexisting risk for suicide; specifically, differentiating between couples with a history of suicide attempts, suicidal thoughts only, and controls (i.e., no previous suicide attempts or ideation). Negative communication behaviors, positive communication behaviors, cognitive bias, implicit relationship satisfaction, entrainment, and feature-derived markers likely comprise a spectrum of risk, such that couples with a history of suicide attempt demonstrate the relative greatest degree of pathology on all these variables, control couples demonstrate the relative lowest degree of pathology, and couples with a history of suicide ideation fall somewhere in between these two extremes. 

Because suicide prevention requires the ability to accurately measure change in suicide risk over time, especially change from suicidal thought to action, a second (and more urgent) empirical test is the ability to detect and predict indications of fluctuations in suicide risk. More specifically, a combination of behavioral and cognitive markers must be able to predict the likelihood and timing of future suicide attempts. It currently remains unclear which combination of behavioral and cognitive features are likely to be the most useful for detecting escalations in suicide risk. In the absence of this knowledge, a logical starting point is to use the same set of candidate markers delineated for the first empirical test. However, it is important to note that different sets of markers may be useful for distinguishing amongst risk groups characterized by historical difference in suicidal behaviors and risk groups characterized by their risk of suicide behaviors in the near future. Thus, examination of predictors of short-term fluctuations in future suicide risk should not be limited to markers associated with existing risk group membership.

The final empirical test for BSP and computer-assisted tests of cognitive bias is an evaluation of their robustness in passing tests 1 and 2 using data collected under different circumstances characterized by differing levels of data quality. Indeed, one especially promising aspect of these methods is their potential to be accessed and used in nonclinical settings, to include availability at home via the internet or other personally-owned devices (e.g., smart phones, tablets, computers). To date, however, BSP and computer-assisted tests of cognitive bias have largely been implemented in highly-controlled laboratory or health care settings. Data collected under such conditions are likely to differ from data collected in less controlled environments such as the home or workplace. For example, data collected in non-laboratory settings are more likely to contain additional artifacts such as background noise, interruptions, external distractions, and/or failure to complete the whole task. Furthermore, consumer-grade audio- and video-recording devices that are likely to be owned and used by individuals tend to generate a much lower quality recording than the carefully-calibrated, professional-grade microphones and cameras that are often used in research settings. Likewise, the precise measurement of timing required by computer-administered cognitive tests may be impacted by variable internet connection speeds. These difference could impact the robustness of these methods to detect suicide risk, thereby limiting their practicality and potential impact. In order to achieve their full potential, additional information about the performance of BSP and computer-based methods must be assessed under the noisy, incomplete, and imprecise conditions that characterize data collected outside of laboratory and healthcare settings.

## 8. Testing the Safety and Minimizing Possible Iatrogenic Effects of BSP-Based Measures of Behavior and Computer-Based Measures of Implicit Cognitive Bias to Detect Risk for Suicide

In addition to conducting tests of the ability of BSP- and computer-based measures to detect risk for suicide, it would also be vital to ensure that these methods do not have unintended, iatrogenic side effects that result in increased risk for suicide. There is strong empirical reason to think that the likelihood of iatrogenic effects is very low. For example, a large body of work in relationship science is based on human coding of conversations between spouses (see [36] for a review). This work has been conducted in a wide range of normative and disordered populations including well-functioning community couples [30], couples where one partner has had an affair [75], couples where one or both partners have perpetrated intimate partner violence [76], couples where one or both partners have been diagnosed with a mood [37] or substance abuse [77] disorder, and couples where one or both partners is a military veteran and has symptoms of Post-traumtic Stress Disorder [78]. There is no empirical evidence that discussing relationship difficulties has resulted in enduring increases in relationship dysfunction, psychological distress, or psychological symptoms following participation in these studies (e.g., [79]). A small number of participants (e.g., 3%–5%) report that they learned something new about their partner or their relationship during these discussions that was temporarily upsetting, but none of these participants reported an unwillingness to participate in similar research in the future as a result of this temporary distress. However, given that relationship conflict is the most commonly reported interpersonal stressor that precedes suicide attempts and that acute relationship flare-ups may last longer and be harder to resolve for chronically distressed couples, it is extremely important that the impact of discussing relationship issues on an at-risk National Guard personnel’s suicidal ideation be directly and thoroughly assessed before such methods could be considered for wide-spread implementation.

Though we anticipate that talking with one’s romantic partner will not have iatrogenic effects, there are several procedures that could be used to minimize risk for iatrogenic effects. These procedures draw on well-established and widely used methods in the broader relationship science literature [79] as well as techniques from empirically established couple therapies, such as Integrative Behavioral Couple Therapy [80]. First, we recommend that both partners discuss any concerns that they may have about talking about relationship issues prior to a discussion of relationship issues. If either spouse is uncomfortable talking about relationship issues for the purposes of assessing risk for suicide, this discomfort should be respected and these couples should strongly consider not discussing relationship issues at that point in time. Such couples may consider completing a computerized performance test of cognitive bias instead of discussing relationship issues. If both partners are comfortable discussing relationship issues, a future time should be agreed on for discussing relationship issues. Establishing a future time for the discussion is important because it allows both partners to think about what they would like to say. It additionally allows both partners to clear up their schedules so that the discussion does not occur during a time when they anticipate feeling stressed or overwhelmed (e.g., it does not occur when one or both partners feels like they need to be doing something else like work for their job(s)). Third, both partners should agree on the specific relationship issues to be discussed in advance. Agreeing on the topics to be discussed minimizes uncertainty about what the other partner might bring up and allows partners to reflect on how discussion of these topics has occurred in the past. Additionally, these topics should be of moderate concern at most and should not be topics that typically result in explosive arguments. Both partners should feel highly confident that the topics can be discussed without creating harm in their relationship or high levels of distress in one another. Fourth, partners should agree on a set period of time for the discussion. Research in relationship science recommends that discussions last approximately 10 to 15 min. This period of time allows for a sufficient sample of behavior to reliably assess relationship dynamics while also minimizing the amount of time spent discussing potentially upsetting topics. Fifth, partners should agree to postpone the planned conversation if they are feeling upset with or angry at one another immediately prior to the time for the planned conversation. Finally, partner should agree on a plan for what to do if either of both of them experiences distress during the discussion. 

## 9. Conclusions

BSP-based measurement of behavioral markers and computer-administered cognitive tests of implicit cognition have the potential to be used to obtain a more precise assessment of a soldier’s risk for suicide by overcoming two key limitations of existing methods for assessing suicide risk: requiring direct and accurate disclosure of psychological distress, and limited accessibility outside of healthcare (and research) settings. By circumventing these two limitations, BSP and computer-administered methods are well-suited to providing military couples with a new set of tools designed to safeguard at-risk soldiers. Before the utility and practicality of these methods can be fully realized, however, these methods must undergo several rigorous empirical tests.

## Figures and Tables

**Figure A1:**
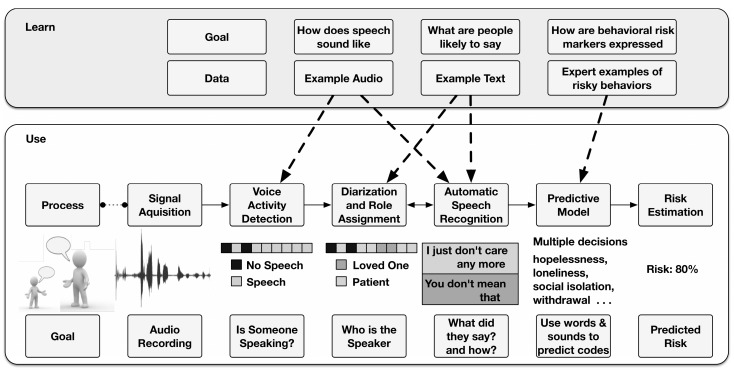
Steps involved in BSP analysis of behavioral markers of suicide risk.

## Appendix A

Before audio recordings can be used to generate automatic observational coding markers and feature-derived behavioral markers, there are several data processing steps that need to be completed (see Figure A1). These steps include Voice Activity Detection (i.e., determining when sound is speech versus from some other source) and diarization (i.e., determining who is talking when). Below, we provide a technical introduction to these data processing steps.

### ***Voice Activity Detection*** ***(VAD)***

The first processing step in preparing a recorded conversation for feature extraction is to determine when the acoustic signal contains spouses’ speech vs. when it contains only ambient/background noise or silence. It is neither economical nor feasible to perform this task (i.e., marking speech boundaries) manually. Even relatively cheaper crowd-sourcing techniques are still costly and privacy issues and turn-around time make them impractical alternatives for processing recordings of spouse-Soldier conversations.

Due to the critical role of VAD in so many speech processing applications, researchers have long focused on the problem and proposed several signals features for separating speech and non-speech segments. These features include short-term information ranging from 10 ms to 40 ms from energy-based features (e.g., [81]), zero-crossing rate (e.g., [82]), wavelet-based features (e.g., [83]), correlation coefficients (e.g., [83]) and negentropy [84], which has been shown to perform well in low signal-to-noise ratio environments. Long-term features in the range of 50–100 ms (e.g., [85]) and above 150 ms (e.g., [86]) have been shown to perform well on noisy speech conditions under a variety of environmental noises; they also capture information that short-term features lack. Other methods of VAD rely on first assuming speech presence throughout the signal and deriving speech presence features from an automatic speech recognition system output. This approach has the advantage that it can take lexical structure into consideration and hence perform better under certain conditions (e.g., [87]).

### ***Diarization***

The second processing step in preparing a recorded conversation for feature extraction is to identify when the Soldier is speaking, the spouse is speaking, or some other person is speaking (as may be the case for recordings made at home). Diarization uses acoustic features to divide a signal that contains multiple speakers into several signals that each contain a single speaker and answers the question of “who speaks when”.

Research on diarization methods can be summarized into three groups according to the procedure. The first and main kind of methods adopt a “bottom-up” approach which merge small audio segments agglomeratively into larger clusters corresponding to individual speakers (e.g., [88]). The second kind of methods handle the problem from a “top-down” direction which treat the entire signal as from one speaker and then add speakers iteratively (e.g., [89]). The third kind of methods attempt to learn the speaker characteristics by iteratively decoding the speaker and re-training the model based on the hypothesized speaker identity (e.g., [90]). In terms of mathematical models, various statistical and machine-learning methods are applied to diarization problems. For example, classical methods use Gaussian Mixture Model to represent the acoustic characteristics of each speaker and K-means algorithms to cluster the speech segments (e.g., [91]). Statistical methods such as Bayesian Information Criterion (e.g., [92]), Generalized Likelihood Ratio (e.g., [93]), Kullback-Leibler divergence (e.g., [94]), and Information Change Rate (e.g., [95]) are used for both segmenting and clustering the speech. Lastly, machine learning methods such as Variational Bayes (e.g., [96]), Non-parametric Bayes (e.g., [97]), and Factor Analysis (e.g., [98]) are introduced to solve the diarization problem.
